# Ramelteon ameliorated 1-methyl-4-phenylpyridinium (MPP+)-induced neurotoxicity in neuronal cells in a mitochondrial-dependent pathway

**DOI:** 10.1080/21655979.2021.1960767

**Published:** 2021-08-04

**Authors:** Chuo Li, Yusheng Zhang, Rongrong Liu, Yuzhen Mai

**Affiliations:** a Department of Neurology, Guangzhou Eighth People’s Hospital of Guangzhou Medical University, Guangzhou, Guangdong, China; b Department of Neurology and Stroke Center, The First Affiliated Hospital of Jinan University, Guangzhou, Guangdong, China

**Keywords:** Parkinson’s disease, mpp+, mitochondria, ramelteon

## Abstract

Parkinson’s disease (PD) is a common neurodegenerative disease with global health and economic impact. 1-methyl-4-phenylpyridinium (MPP+)-induced mitochondrial dysfunction and oxidative stress are reported to participate in the pathological mechanism of PD. Ramelteon is a novel oral hypnotic agent that has recently been reported to display neuronal protective effects. However, it is unknown whether Ramelteon possesses a beneficial effect in PD. In this study, we aimed to examine the potential function of Ramelteon in MPP+-challenged neurons. We found that Ramelteon rescued the cell viability reduced by MPP+-stimulation. Further, oxidative stress in MPP+-challenged SH-SY5Y cells was mitigated by Ramelteon as verified by the upregulated levels of mitochondrial reactive oxygen species (ROS) and protein carboxyl, and the upregulation of NADPH oxidase 4 (NOX-4). Furthermore, the declined mitochondrial membrane potential (_Δ_Ψ_m_) caused by MPP+ was reversed by Ramelteon. Importantly, Ramelteon attenuated MPP+-induced apoptosis, accompanied by a decreased ratio of Bax/Bcl-2, inhibition of cytochrome C release, and downregulation of cleaved caspase-3. For the first time, we conclude that Ramelteon might ameliorate MPP+-induced neurotoxicity in neuronal cells in a mitochondrial-dependent pathway.

## Introduction

Parkinson’s disease (PD) has a morbidity of 1.7% among the population over 65 years old [1]. The main clinical observations of PD include static tremor, myotonia, bradykinesia, postural and gait abnormalities, olfactory disorders, anxiety and depression, cognitive decline, sleep disorders, obstacles, autonomic nervous dysfunction, pain, and fatigue [2,3]. Loss of dopaminergic neurons and formation of Lewy bodies in the dense part of the midbrain substantia nigra are both main pathological characteristics of PD [4]. The pathogenesis of PD involves several complex mechanisms. Recently, oxidative stress has been linked to the pathological mechanism of PD. Reactive oxygen species (ROS) are produced from exogenous oxidants or intracellular aerobic metabolic processes, such as superoxide anions, hydrogen peroxide, hydroxyl radicals, the production and metabolism of which are maintained by the oxidative system and the anti-oxidative system [5,6]. However, when the secretion of ROS exceeds the physiological demands, the cellular structure and function are influenced due to the oxidative degradation of DNA, proteins, and lipids, resulting in oxidative stress [7]. The involvement of ROS in the pathogenesis of PD has been well reported. Malondialdehyde (MDA), an important marker of lipid peroxidation, was significantly upregulated in the intracerebral lesions of PD mice induced with 1-methyl-4-phenyl-1,2,3,6 -tetrahydropyridine (MPTP) [8]. MPTP and MPP+ (an active metabolite of MPTP) were found in the 1970s. Previous studies have reported that MPTP/MPP+ has a powerful effect on inducing PD in both *in vivo* and *in vitro* models [9]. A recent study showed that MPP+ injections in rat models caused nigral lesions. MPP+ is also responsible for the formation of Lewy bodies in humans with PD [10]. Excessive production of ROS was observed in the homogenate of cerebral striatum and substantia nigra tissues of PD mice. Also, the expression of GSH, an anti-oxidative substance, was significantly downregulated in the brain tissue of PD mice. The excessive production of ROS is closely related to mitochondrial dysfunction. Mitochondria is a vital power pack that produces adenosine triphosphate (ATP) through oxidative phosphorylation of the electron transport chain, accompanied by the secretion of ROS mediated by NADPH oxidase 4 (NOX-4) (mitochondria enzyme). Subsequently, ROS is catalyzed into divalent oxygen and water by superoxide dismutase (SOD) or glutathione (GSH) located in the mitochondria to protect the cells from oxidative stress damage. It is reported that the electron transport chain enzyme complex is deficient in the mitochondria of PD nigra tissues, which contributes to mitochondrial dysfunction and accumulation of ROS [11]. As a result, oxidative stress is induced, and the apoptosis of neurons is facilitated. Therefore, alleviating oxidative stress by maintaining the function of mitochondria becomes a strategy for PD treatment.

Ramelteon is an oral hypnotic agent developed by Takeda and approved for the treatment of insomnia by the Food and Drug Administration (FDA) in the U.S. in 2015 [12]. As the first agonist of melatonin receptor, Ramelteon showed promising therapeutic effects against acute insomnia and chronic insomnia during clinical treatments [13,14]. Recently, protective effects of Ramelteon against damage induced by oxidative stress have been reported [15,16]. However, whether Ramelteon exerts any protective benefits in PD is still unknown. Here, we used MPP+ to treat SH-SY5Y cells to establish an *in vitro* PD model. We aimed to examine the potential roles of Ramelteon against MPP+-induced toxicity in neuronal cells.

## Materials and methods

### Cell culture and treatments

SH-SY5Y cells (ATCC, California, USA) were incubated in DMEM/F12 with 10% FBS and 5% CO_2_ at 37°C. Cells were stimulated with MPP+ (#D048, purity≥98%, Sigma-Aldrich, USA) (500 µM) with Ramelteon (#SML2262, purity≥99%, Sigma-Aldrich, USA) (50, and 100 nM) for 36 hours.

### Cell counting kit-8 (CCK-8) method

Cells were seeded in a 96-well plate (2 × 10^3^ cells per well), then incubated for 48 hours. Then, 10 μL CCK-8 was pipetted into each well, followed by incubation for 2 hours. Finally, the microplate reader (Thermo, MA, USA) was used to measure the optical density at 450 nm to calculate cell viability.

### Mitosox red staining

The level of mitochondrial ROS was detected using MitoSox red assay (Thermo, Massachusetts, USA). SH-SY5Y cells were seeded in a 24-well plate (5 × 10^4^cells/well) and were further incubated with 5 μM MitoSox Red in the dark for half an hour. After collecting the cells and being washed 3 times using PBS buffer, the red fluorescence was checked using a fluorescent microscope.

### Protein carboxyl detection

The concentration of protein carboxyl in the treated SH-SY5Y cells was determined using ELISA assay according to the instruction described previously. The OD values recorded at 450 nm were used to calculate the concentration of protein carboxyl.

### RH123 staining

The level of_Δ_Ψ_m_ was detected using RH123 staining. Briefly, Rh123 (1 mg/mL in dimethyl sulfoxide) was added, incubated for half an hour. After washing 3 times using PBS buffer, the cells were then collected and analyzed with a fluorescent microscope (excitation: 488 nm; emission: 530 nm).

### Real-time PCR

Total RNA was isolated from SH-SY5Y cells using Qiazol (Qiagen, Germany). The purity and concentration of RNA were assessed using Nanodrop spectrophotometers. Isolated RNA was transcribed into cDNA utilizing the Affinity Script qPCR cDNA Synthesis Kit (Agilent, California, USA), followed by amplifying real-time PCR using SYBR Green Supermax (Bio-Rad, USA) with the following protocol: 50°C (2 minutes), 1 cycle; 95°C (10 minutes), 1 cycle; 95°C (15 seconds), 60°C (30 seconds) and 72°C (30 seconds), 40 cycles; 72°C (10 minutes), 1 cycle. The expression of GAPDH was detected for normalization and the relative expression level of target genes was calculated. The specific primer sequences for Nox4 are: 5’-GCAGGATCCGTCATAAGTCATCCCTCAGA-3’ (forward) and 5’-GCTGTTAACGTCGACTCAGCTGAAAGACTCTTTAT-3’ (reverse) and the primer sequences for GAPDH are: 5’-GACGGCCGCATCTTCTTGT-3’ (forward), and 5’-CAGTGCCAGCCTCGTCCCGTAGA −3’ (reverse).

### Western blot analysis

The total proteins were extracted from the cell lysates, followed by being separated on 12% SDS-PAGE. The samples were then transferred onto the PVDF membrane and incubated with 5% nonfat milk to remove the nonspecific sites. Then, the membrane was incubated with primary antibodies (Cell Signaling Technologies, Boston, USA) against NOX-4 (1:2000), Bax (1:1000), Bcl-2 (1:1000), cytochrome C (1:1000), cleaved Caspase-3 (1:500), or β-actin (1:10,000) overnight at 4°C, followed by being incubated with appropriate secondary antibodies (1:100, Cell Signaling Technologies, Boston, USA) for 1.5 hours at room temperature. ECL system (Pierce, Illinois, USA) was used to assess the relative expression of the target proteins [17].

### Fragmentation of cytosol and mitochondria

Mitochondrial and cytosolic fractions were isolated through resuspending the cells in buffer A (225 mM mannitol, 75 mM sucrose, 2 mM 126 K_2_PO_4_, 0.1% BSA, 5 mM Hepes, 1 mM EGTA (pH 7.2)) on ice. After lysing the cells with a cell homogenizer (Thermo, Massachusetts, USA), homogenates were pelleted at a condition of 10 minutes and centrifuged at 750 × g, and the supernatant was collected and subjected to another centrifugation at 10,000 × g for 30 minutes. The precipitation was suspended using buffer A, which was the mitochondrial part. The generated supernatant was then centrifuged at 100,000 × g for 1 hour, and the final supernatant represented the cytosolic fractions.

### TdT-mediated dUTP Nick-End Labeling (TUNEL) staining

The treated SH-SY5Y cells were planted in poly-l-lysine-treated coverslips, which were stained with TUNEL (Thermo Fisher Scientific, Massachusetts, USA) according to the instruction described previously, with the cell nuclei mounted with DAPI. The TUNEL-positive cells were counted for quantification under a fluorescence microscope (Olympus, Tokyo, Japan) [18].

### Statistics

Data were analyzed using ANOVA or Mann-Whitney using Prism 5.0 software. p < 0.05 were considered statistically significant.

## Results

Here, our findings demonstrate a novel function of Ramelteon by showing that Ramelteon protects SH-SY5Y neuronal cells against MPP+-induced oxidative stress, mitochondrial dysfunction, and apoptosis.

### Ramelteon ameliorates MPP+-induced decrease in cell viability in human SH-SY5Y cells

**Figure 1.**
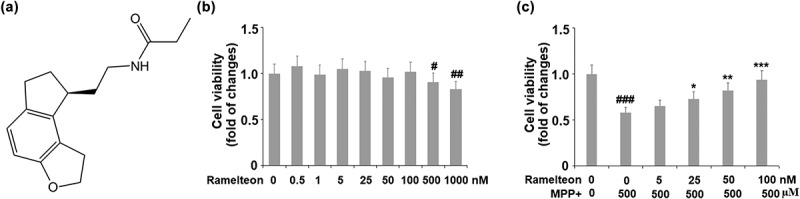
Ramelteon ameliorated MPP+-caused decreased cell viability in human SH-SY5Y cells. (a). Molecular structure of Ramelteon; (b). The effects of Ramelteon alone in cell viability of human SH-SY5Y cells. Cells were cultured with Ramelteon alone at the concentrations of 0, 0.5, 1, 5, 25, 50, 100, 500, and 1000 nM for 36 hours. (c) Cells were stimulated with MPP+ (500 µM) with or without Ramelteon (5, 25, 50, and 100 nM) for 36 hours. Cell viability was measured using CCK-8 (#, ##, ###, P < 0.05, 0.01, 0.005 vs. vehicle group; *, **, ***, P < 0.05, 0.01, 0.005 vs. MPP+ group, N = 5–6)

To screen the safe incubation concentrations of Ramelteon, cells were incubated with Ramelteon alone at the concentrations of 0, 0.5, 1, 5, 25, 50, 100, 500, and 1000 nM for 36 hours. No significant difference was found in the cell viability as the concentration of Ramelteon increased from 0 to 500 nM. It was, however, dramatically decreased as the concentration of Ramelteon increased to 500 and 1000 nM (Figure 1b) (#, ##, p < 0.05, 0.01). Therefore, 50 and 100 nM Ramelteon were used in the subsequent experiments. Then, the cells were stimulated with MPP+ (500 μM) with or without Ramelteon (50 and 100 nM) for 36 hours. As shown in Figure 1c, the cell viability was decreased greatly by MPP+ (###, P < 0.005) but dramatically elevated by Ramelteon (*, **, ***, P < 0.05, 0.01, 0.005), implying that the cytotoxicity in SH-SY5Y cells induced by MPP+ could be alleviated by Ramelteon.

### Ramelteon alleviates oxidative stress in MPP+-challenged cells

**Figure 2.**
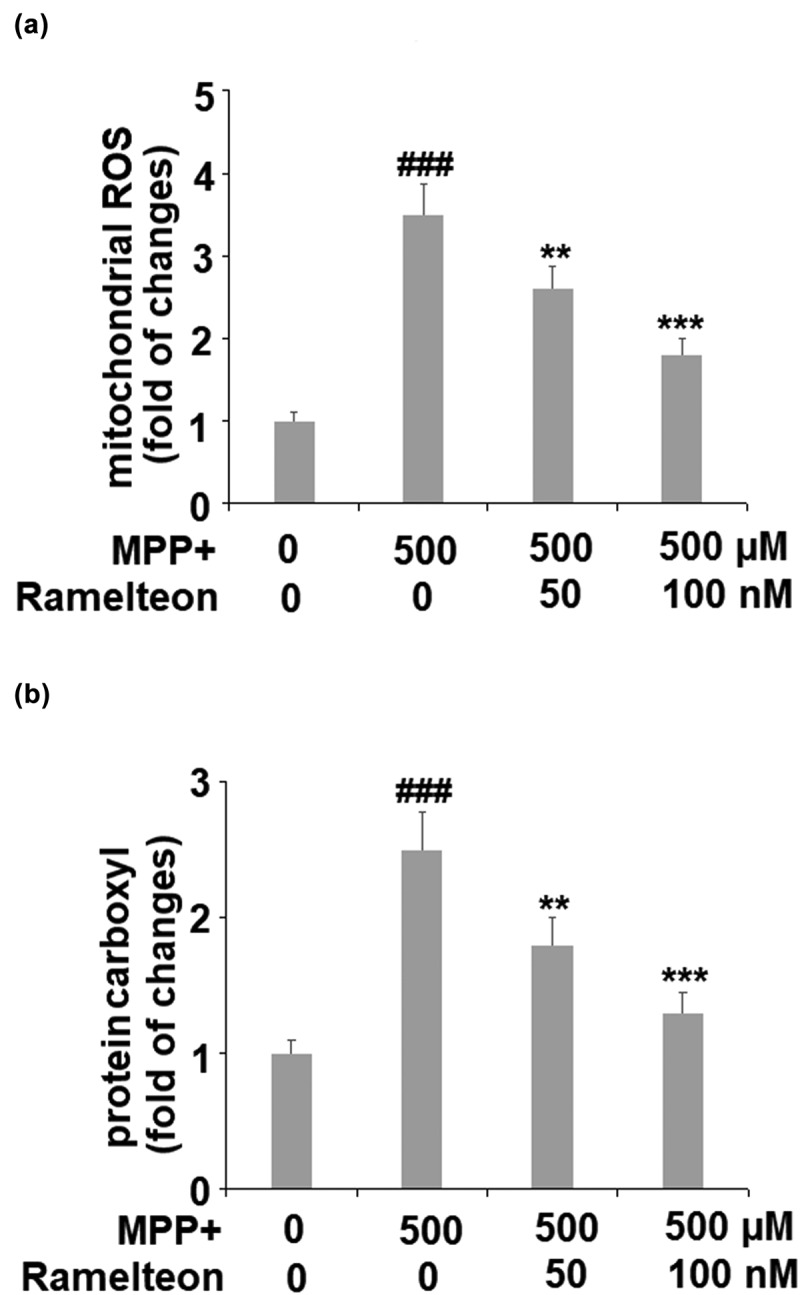
Ramelteon alleviated MPP+-induced oxidative stress. (a). The levels of mitochondrial ROS; (b). Levels of protein carboxyl (###, P < 0.005 vs. vehicle; **, ***, P < 0.01, 0.005 vs. MPP+, N = 5–6)

Mitochondrial ROS were remarkably increased 3.5- fold by MPP+ stimulation (###, P < 0.005), compared to the control. However, the levels of mitochondrial ROS were apparently suppressed to 2.6- (**, P < 0.01) and 1.8- fold (***, P < 0.005) by treatment with 50 and 100 nM of Ramelteon, respectively (Figure 2a). Similarly, the level of protein carboxyl was inhibited to 1.8- (**, P < 0.01) and 1.3- fold (***, P < 0.005), by the same doses of Ramelteon, compared to a 2.5- fold increase (###, P < 0.005) induced by MPP+ stimulation (Figure 2b). These results suggest that oxidative stress in MPP+-stimulated cells was attenuated by Ramelteon.

### Ramelteon reduces NOX-4 in MPP+-treated cells

**Figure 3.**
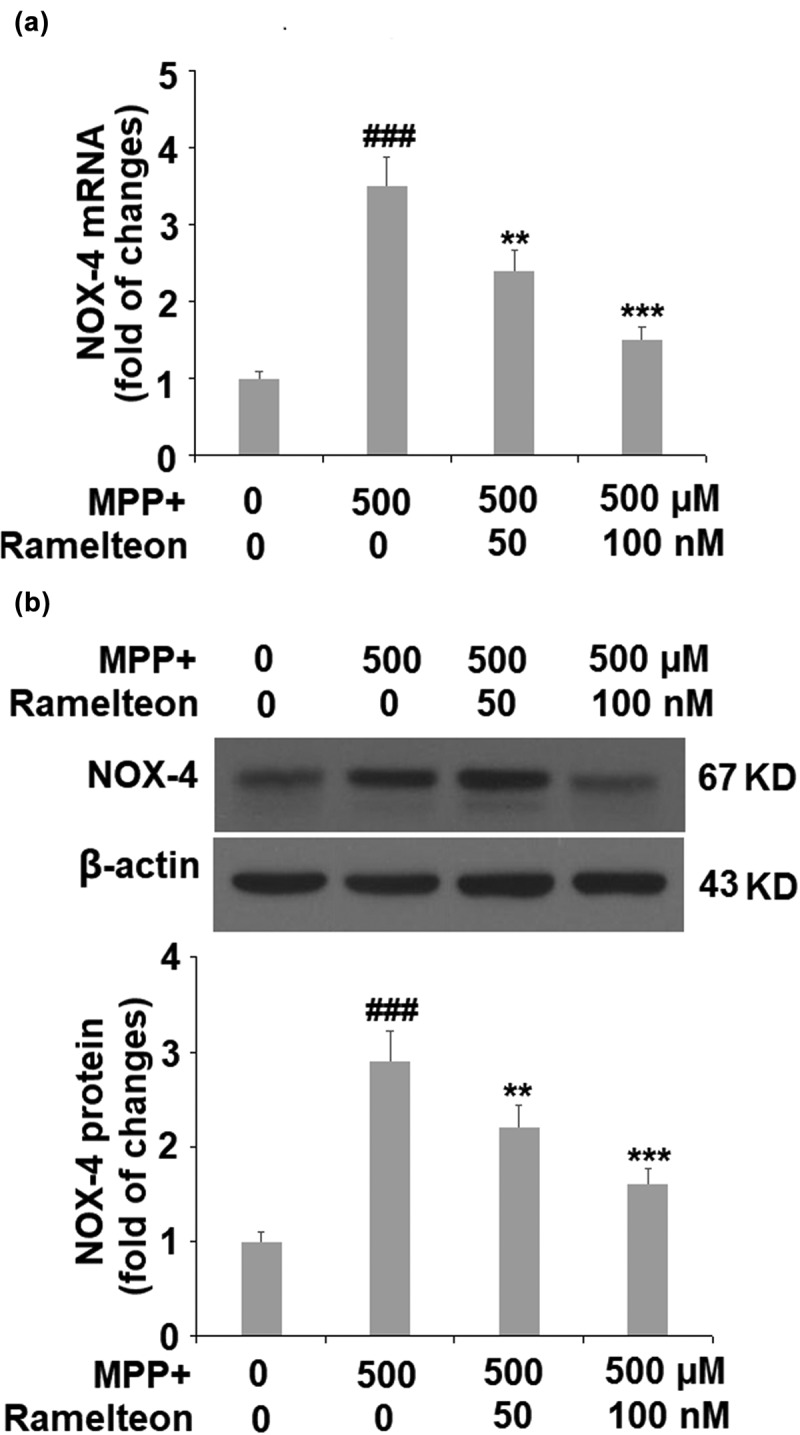
Ramelteon reduced MPP+-caused upregulation of NOX-4. (a). mRNA of NOX-4; (b). Protein level of NOX-4 (###, P < 0.005 vs. vehicle; **, ***, P < 0.01, 0.005 vs. MPP+, N = 5)

Then we investigated the level of the mitochondria enzyme, NOX-4. The mRNA level of NOX-4 was found to be significantly upregulated 3.5-fold by MPP+ (###, P < 0.005), but dose-responsively reduced to 2.4- (**, P < 0.01) and 1.5- fold (***, P < 0.005) by Ramelteon treatment (Figure 3a). Then we measured the protein level of NOX-4, and the protein level of NOX-4 was increased by MPP+ stimulation 2.9- fold (###, P < 0.005), which was suppressed to 2.2- (**, P < 0.01) and 1.6- fold (***, P < 0.005) by the two doses of Ramelteon, respectively (Figure 3b).

### Ramelteon prevents mitochondrial dysfunction caused by MPP+

**Figure 4.**
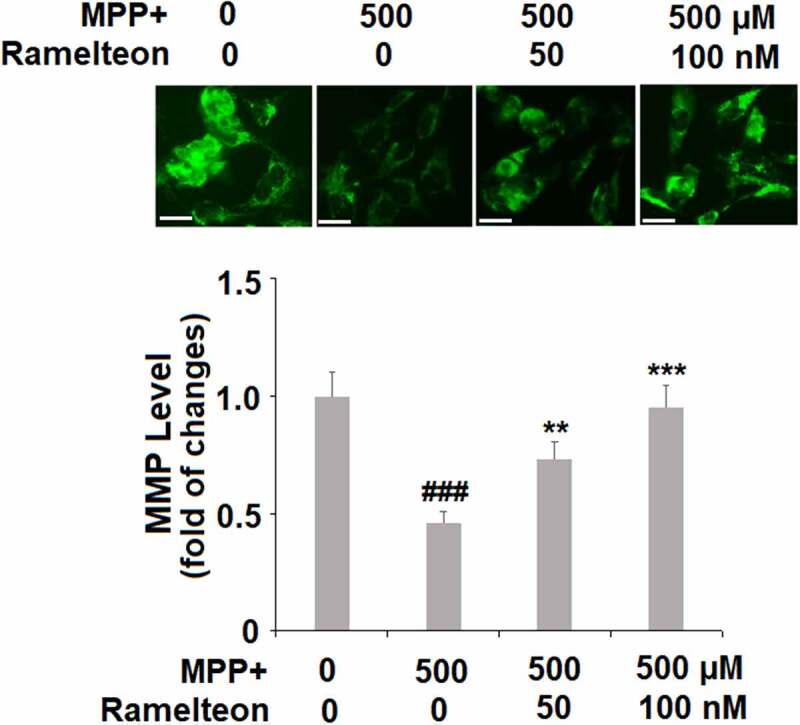
Ramelteon prevented MPP+-induced mitochondrial dysfunction. Cells were stimulated with MPP+ (500 µM) with or without Ramelteon (50, and 100 nM) for 36 hours. Levels of _Δ_Ψ_m_ were examined using RH123. Scale bar, 50 μm. (###, P < 0.005 vs. vehicle; **, ***, P < 0.01, 0.005 vs. MPP+, N = 4–5)

Mitochondrial membrane potential (_Δ_Ψ_m_) is an important marker of mitochondrial function, which was further measured. The _Δ_Ψ_m_ was decreased to 46% (###, P < 0.005) by MPP+-treatment, then rescued to 73% (**, P < 0.01) and 95% (***, P < 0.005) by 50 and 100 nM Ramelteon, respectively, indicating that the mitochondrial function caused by MPP+ was greatly reversed by Ramelteon (Figure 4).

### Ramelteon attenuates apoptosis in MPP+-stimulated cells

**Figure 5.**
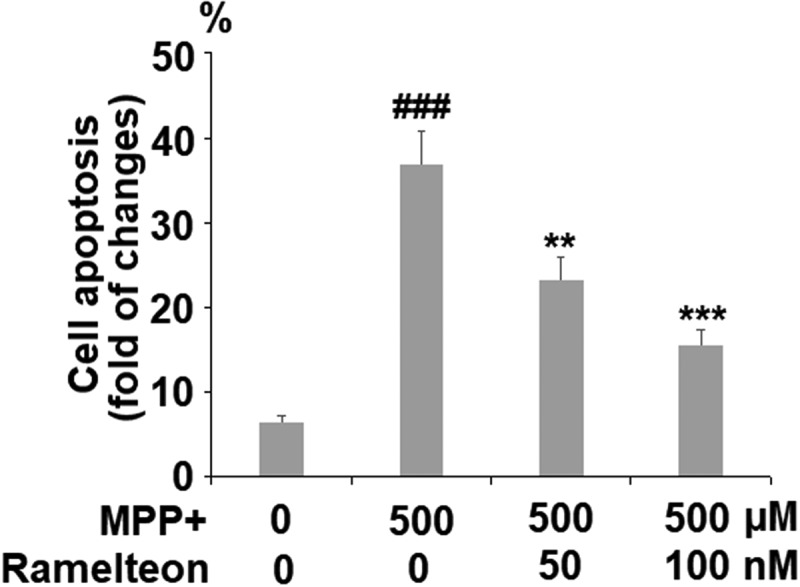
Ramelteon attenuated MPP+-induced apoptosis. Cells were incubated with MPP+ (500 µM) or Ramelteon (50, and 100 nM) for 36 hours. Cell apoptosis (###, P < 0.005 vs. vehicle; **, ***, P < 0.01, 0.005 vs. MPP+, N = 5) was determined by TUNEL

**Figure 6.**
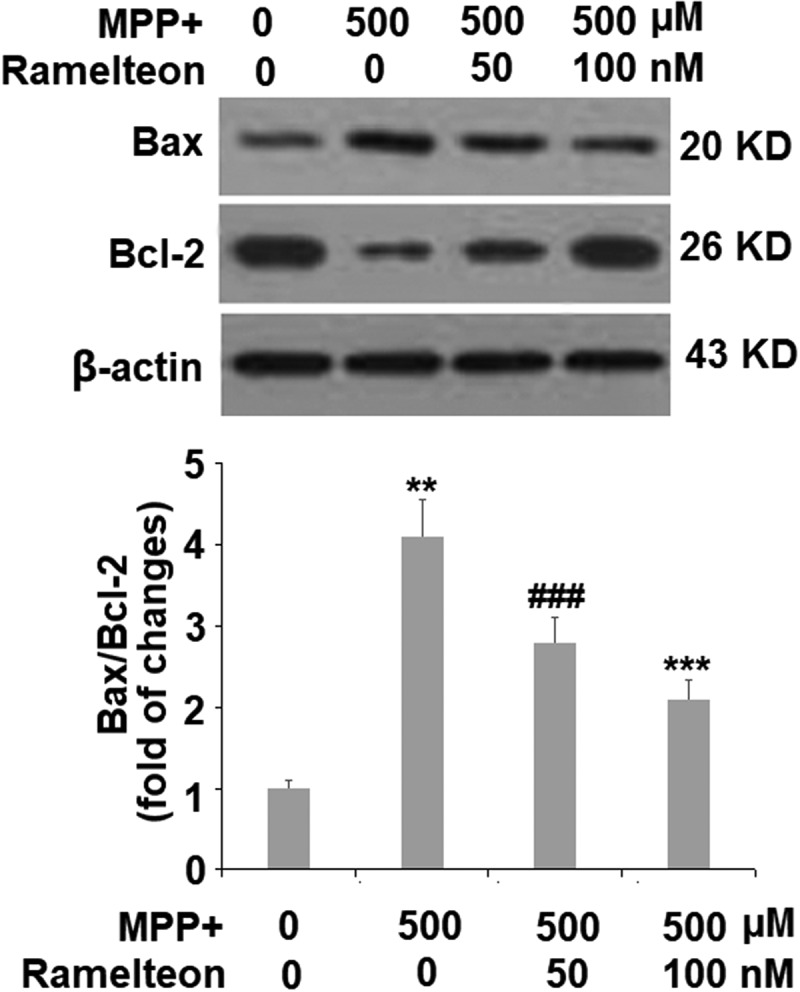
Ramelteon resorted MPP+-induced increase in the ratio of Bax/Bcl-2 Proteins of Bax and Bcl-2 were measured and the ratio of Bax/Bcl-2 was determined (###, P < 0.005 vs. vehicle; **, ***, P < 0.01, 0.005 vs. MPP+, N = 5)

The apoptosis of neurons has been regarded as one of the main pathological characteristics of PD. The apoptotic rate was elevated from 6.5% to 36.8% by stimulation with MPP+ (###, P < 0.005) but was reversed to 23.3% (**, P < 0.01) and 15.6% (***, P < 0.005) by treatment with 50 and 100 nM Ramelteon (Figure 5). In addition, the expressions of Bax and Bcl-2 were determined. The ratio of Bax/Bcl-2 was dramatically increased by stimulation with MPP+ (###, P < 0.005) but pronouncedly decreased by the introduction of Ramelteon (**, ***, P < 0.01, 0.005), indicating a potential neuroprotective property of Ramelteon (Figure 6).

### Ramelteon reduces MPP+-induced release of cytochrome C from mitochondria to cytosol and the level of cleaved of Caspase 3

**Figure 7.**
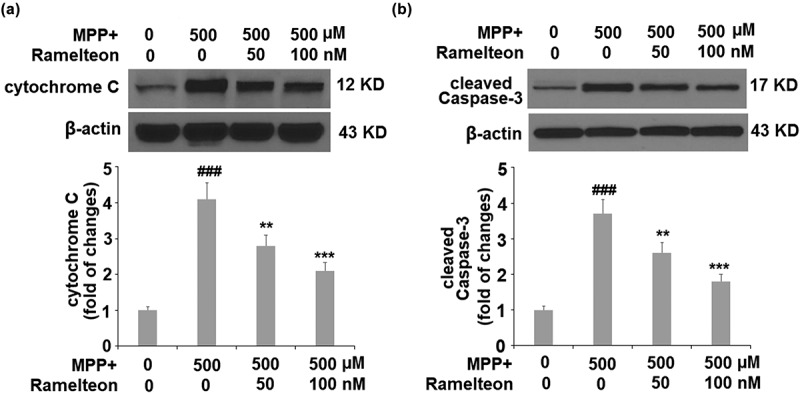
Ramelteon inhibited MPP+-caused release of cytochrome C from mitochondria to cytosol and the level of cleaved Caspase 3 (a). The levels of cytochrome C in the cytosol; (b). The level of cleaved Caspase-3 (###, P < 0.005 vs. vehicle; **, ***, P < 0.01, 0.005 vs. MPP+, N = 5)

To further investigate the effects of Ramelteon on the apoptosis of neurons, we measured the release of cytochrome C from mitochondria to cytosol and the level of cleaved of Caspase 3. The results in Figure 7a show that the level of cytochrome C was significantly increased 3.2- fold (###, P < 0.005) by MPP+-treatment, after which it was reduced to 2.3- (**, P < 0.01) and 1.6- (***, P < 0.005) fold by 50 and 100 nM Ramelteon, respectively. Similarly, the expression of cleaved caspase-3 was also remarkably upregulated 3.7- fold (###, P < 0.005) by MPP+, compared to the control. However, the same doses of Ramelteon reduced the expression of cleaved caspase-3 to 2.6- (**, P < 0.01) and 1.8- fold (***, P < 0.005), respectively (Figure 7b).

## Discussion

It is reported that MPTP can induce the decreased secretion of dopamine, triggering the inactivation of dopamine signaling and contributing to the development of PD-like symptoms. MPP+, a metabolite of MPTP in the astrocytes, is one of the main constituents that induce the excessive secretion of ROS by inactivating respiratory complex II, which eventually results in mitochondrial dysfunction, oxidative stress in neurons, and neuronal apoptosis [19,20]. As described in other reports [21,22], MPP+ has been used to induce a PD-like injury in neuronal cells.

Mitochondria are the source of excessively released ROS that results in intracellular oxidative stress [23,24]. In oxidative processing, NADH-quinone oxidoreductase is the entrance for electrons that are transferred from the mitochondrial matrix into the electron transport chain and are the main sites that produce mitochondrial ROS, induced by decreased ATP production, increased NADH/NAD+ ratio, and reduced coenzyme Q [23]. NOX-4 is one of the main synthesis enzymes for the production of ROS located in mitochondria [25]. When mitochondrial dysfunction is induced by external factors, excessive production of ROS and oxidative stress are triggered, contributing to the dysfunction of mitochondria and cell apoptosis [26].

MPP+-challenged SH-SY5Y cells showed a declined cell viability, reduced level of mitochondrial membrane potential, and increased apoptotic rate. By treatment with Ramelteon, these pathological changes were significantly reversed. In our experiment, an oxidative stress state was significantly activated by stimulation with MPP+, which was verified by the elevated level of mitochondrial ROS and protein carboxyl, as well as the upregulation of NOX-4. By the administration of Ramelteon, oxidative stress in MPP+-stimulated SH-SY5Y cells was dramatically alleviated, indicating its potential protective effect of Ramelteon against oxidative stress-induced injury to neurons.

Recent progress reported that the key process of apoptosis is not located in the nuclei but the cytoplasm. Before characteristic morphological changes and DNA degradation induced by apoptosis, the function of the mitochondrial membrane is impaired, including the disappearance of endometrial transmembrane potential and the release of protease activators from mitochondria, which mediate the apoptosis-related metabolic changes [27,28]. Here, we found that apoptosis of SH-SY5Y cells was significantly induced by MPP+, accompanied by the declined _Δ_Ψ_m_ and the loss of cytochrome C, indicating that MPP+- induced mitochondrial apoptosis processing. The mitochondrial apoptosis process was suppressed through treatment with Ramelteon, indicating a protective effect of Ramelteon on the key process of apoptosis. The Bcl-2 family is an essential apoptosis-related gene family that mediates the process of apoptosis by governing the permeability of the mitochondrial outer membrane to control the release of proteins in mitochondria [29]. Bax is a pro-apoptotic protein in the Bcl-2 family. The ratio of Bax/Bcl-2 is a useful index for determining apoptotic state [30]. The anti-apoptotic property of Ramelteon was further confirmed by the decreased Bax/Bcl-2 ratio and the downregulation of cleaved caspase-3, another pro-apoptotic protein [31].

In recent years, Ramelteon has been trialed to treat insomnia and improve sleep disorders in PD [32]. In preclinical studies, long-term administration of Ramelteon improves post-traumatic stress disorder (PTSD)-like behaviors in genetic mice. The most recent publication shows that oral administration of Ramelteon attenuated brain ischemic injury in an experimental ischemia mouse model [33]. Besides, in an *in vitro* PD model, Ramelteon exerts protective effects on LPS-induced inflammatory response in the brain by inhibiting microglia activation [34]. Furthermore, it has been shown that Ramelteon also offers neuroprotective benefits in Alzheimer’s disease (AD) and Huntington’s disease (HD) [35]. As a melatonin agonist, Ramelteon also shows its capacity to protect against neurotoxic effects in the development of AD. Other *in-vitro* studies report that several biological pathways are affected when neuronal cells are treated with Ramelteon. Previous studies report that Ramelteon modulates the anti-oxidative regulator nuclear factor E2 related factor 2 (Nrf2) [15] and the nuclear factor kappa-B (NF-κB) [36] pathway. Ramelteon also activates the neuronal ERK1/2pathway[37]and AMPK/mTOR signaling pathway. It also stimulates the expression of neuronal Brain-derived neurotrophic factor (BDNF), which is identified as a neuronal survival factor related to brain memory [38]. Our study provides ample evidence that Ramelteon has neuroprotection against neurotoxic MPP+-induced mitochondrial dysfunction and neuronal apoptosis. Therefore, Ramelteon could have a broad range of regulation in neuronal tissues. In our future work, we will study Ramelteon’s specific target, such as Nrf2 signaling, that mediates the function of Ramelteon on oxidative stress in order to better understand the mechanism of Ramelteon’s therapy on neuron injuries induced by oxidative stress. Also, the therapeutic property of Ramelteon against PD will be pursued in the PD animal model established by stimulation with MPTP, accompanied by an evaluation of neurological scores and pathological scores on brain tissue.

Collectively, for the first time, our study shows that the hypnotic agent Ramelteon possesses a protective effect against MPP+-induced neurotoxicity. Ramelteon shows a dose-responsive rescue effect on MPP+-induced neuronal cell cytotoxicity. Mechanistically, Ramelteon alleviates MMP+-induced cellular oxidative stress and mitochondrial membrane potential alternation. Ramelteon shows an anti-apoptotic effect by reducing Bax/Bcl-2 ratio, suppressing cytochrome C release and caspase-3 cleavage.

## Conclusion

Our data concludes that Ramelteon possesses the neuronal benefit against MPP+-induced neurotoxicity in a mitochondrial-dependent pathway.
